# Complete genome sequence of *mcr-9* containing *Leclercia adecarboxylata*

**DOI:** 10.1128/MRA.00481-23

**Published:** 2023-08-14

**Authors:** William Hutton, Ellie Allman, Claudia McKeown, Andrew C. Singer, Adam P. Roberts

**Affiliations:** 1 Department of Tropical Disease Biology, Liverpool School of Tropical Medicine, Liverpool, United Kingdom; 2 UK Centre for Ecology and Hydrology, Wallingford, United Kingdom; DOE Joint Genome Institute, Berkeley, California, USA

**Keywords:** coliston resistance, berberine, berberine resistance, flavanoid

## Abstract

Here, we provide the genome sequence of a *Leclercia adecarboxylata* isolated from a screen of an environmental bacterial isolate library for resistance to the plant flavonoid berberine. We detected the colistin resistance gene *mcr-9*, located on an IncFII(pECLA) plasmid.

## ANNOUNCEMENT

*Leclercia adecarboxylata* was first described as *Escherichia adecarboxylata* in 1962 and subsequently reclassified (1). They are Gram-negative opportunistic pathogens found within the Enterobacteriaceae family, isolated from both clinical (2, 3) and environmental sources (4 - 7).

We isolated *Leclercia* sp. after screening the “Swab and Send” bacterial isolate library (8) for tolerance to 1,000 µg/mL of berberine, an antimicrobial plant flavonoid (9), in Muller-Hinton broth (MHB) at 37°C for 24 h without agitation. For short-read sequencing, genomic DNA extraction and genome sequencing were carried out by MicrobesNG (http://microbesNG.com). Genomic DNA was extracted according to MicrobesNG Genome Sequencing Methods protocol (v20230314), and libraries were prepared using Nextera XT Library Prep Kit (Illumina, San Diego, USA). Sequencing was performed using NovaSeq 6000 Illumina sequencing platform (2 × 250 bp paired-end reads). Trimming and quality filtering of reads were also carried out by MicrobesNG using Trimmomatic (v0.30) with a sliding window quality cutoff of Q15. Trimmed short-reads had an N50 of 251 bp and a read depth of 63× as determined by dividing bases of trimmed and assembled reads (309,836,606/4,884,641).

For long-read sequencing, genomic DNA was extracted from 10 mL overnight cultures grown in MHB broth at 37°C with 200 rpm agitation, using Fire Monkey High Molecular Weight DNA Extraction Kit (RevoluGen). Preparation of libraries was carried out using the Oxford Nanopore Technologies ligation sequencing kit (SQKLSK109) and native barcoding expansion kit (EXP-NBD104). *L. adecarboxylata* (SAS216F10) was assigned to native barcode 4 (NBD04). Long-read sequencing was performed using the R9.4.1 flow cell for 72 h. Reads were basecalled using a high-accuracy basecalling mode and demultiplexed using Guppy (v6.2.1). This resulted in 206,069,063 bp in 16,368 raw reads, with an N50 of 23,820 bp and a read depth of 42×. For *de novo* assembly, barcoded long-read sequences were trimmed using Porechop (v0.2.4; https://github.com/rrwick/Porechop). Trimmed reads were then filtered using Filtlong (v0.2.1; https://github.com/rrwick/Filtlong) with a minimum read length of 1 kbp and a kept-base percentage of 90%. Long-read sequences were assembled using Flye (v2.8.3-b1695) (10), followed by long-read polishing of the draft assembly using Medaka (v1.5.0; https://github.com/nanoporetech/medaka). The draft assembly was then polished with short-read sequences using Polypolish (v0.5.0) (11), followed by further short-read polishing using POLCA (from MaSuRCA v4.0.7) (12). Default parameters were used for all programs unless otherwise stated. GC content (%) and contig size (bp) of the final *de novo* assembled genome were determined using GeneiousPrime (v2023.0.4). ResFinder (v4.1) (13) was used to identify antimicrobial resistance genes, and Mobile Element Finder (v1.0.3) was used to identify mobile genetic elements (14).

The assembly resulted in three contigs, a chromosome (4,673,583 bp; GC content of 55.8%), one IncFII(pECLA) plasmid (187,652 bp; GC content of 49.3%), and another plasmid (23,406 bp; GC content of 56.4%). *L. adecarboxylata* SAS216F10 was originally recovered from a swab of a horse brush. Analysis of the genome shows it contains one copy of the colistin resistance gene *mcr-9*, located on the IncFII(pECLA) plasmid. Mcr-9 has been previously demonstrated to be required for colistin resistance in *Proteus vulgaris* (15). The plasmid located *mcr-9* is associated with the insertion sequence IS*Ehe3* within the IncFII(pECLA) plasmid replicon.

## Data Availability

The genome of *Leclercia adecarboxylata* (SAS216F10) has been deposited in NCBI GenBank under the accession number JASKYL000000000. Paired Illumina short reads (SRR24904737) and ONT long reads (SRR24904736) are publicly available from NCBI SRA Run Selector under BioProject number PRJNA976212.
